# Sequela of female genital mutilation on birth outcomes in Jijiga town, Ethiopian Somali region: a prospective cohort study

**DOI:** 10.1186/s12884-018-1937-4

**Published:** 2018-07-20

**Authors:** Kiros Gebremicheal, Fisehaye Alemseged, Haimanot Ewunetu, Daniel Tolossa, Abdibari Ma’alin, Mahlet Yewondwessen, Samuel Melaku

**Affiliations:** 1 Department of Public Health, Jijiga Health Science College, PO Box 504, Jijiga, Ethiopia; 2 Department of Epidemiology and Biostatistics, College of Public Health and Medical Sciences, Jimma University, PO.Box 1104, Jimma, Ethiopia; 3 Department of Medical Laboratory Technology, Jijiga Health Science College, PO Box 504, Jijiga, Ethiopia; 4 Department of Clinical Nursing, Jijiga Health Science College, PO Box 504, Jijiga, Ethiopia

**Keywords:** FGM, Birth complication, Jijiga town

## Abstract

**Background:**

In Ethiopia, female genital mutilation (FGM) remains a serious concern and has affected 23.8 million women and girls, with the highest prevalence in Somali regional state. Even though FGM is reported to be associated with a range of obstetric complications, little is known about its effects on childbirth in the region. Therefore, the objective of this study was to test the hypothesis that FGM is a contributing factor to the increased risk of complication during childbirth.

**Methods:**

Facility based cohort study, involving 142 parturients with FGM and 139 parturients without FGM, was conducted in Jijiga town from October to December, 2014. The study participants were recruited by consecutive sampling technique. Data were collected using a structured interviewer administered questionnaire and observational checklists. Data were analyzed using SPSS version 16 and STATA version 11.

**Results:**

The existence of FGM was significantly associated with perinealtear [RR = 2.52 (95% CI 1.26–5.02)], postpartum blood loss [RR = 3.14 (95% CI 1.27–7.78)], outlet obstruction [RR = 1.83 (95% CI 1.19–2.79)] and emergency caesarean section [RR = 1.52 (95% CI 1.04–2.22)]. FGM type I and FGM type II did not demonstrate any association with prolonged 2nd stage of labour, emergency caesarean section, postpartum blood loss, and APGAR score < 7. FGM type III however was significantly associated with prolonged 2nd stage of labour [RR = 2.47 (95% CI 1.06–5.76)], emergency caesarean section [RR = 3.60 (95% CI 1.65–7.86)], postpartum blood loss [RR = 6.37 (95% CI 2.11–19.20] and APGAR score < 7 [RR = 4.41 (95% CI, 1.84–10.60)]. FGM type II and type III were significantly associated with perinealtear [RR = 2.45(95% CI 1.03–5.83)], [RR = 4.91(95% CI 2.46–9.77)] and outlet obstruction [RR = 2.38(95% CI 1.39–4.08)], [RR = 2.94(95% CI 1.84–4.71)] respectively.

**Conclusion:**

Women with FGM are significantly more likely than those without FGM to have adverse obstetric outcomes. Risks seem to be greater with more extensive form of FGM. Adverse obstetric outcomes can therefore be added to the known harmful immediate and long-term effects of FGM.

**Electronic supplementary material:**

The online version of this article (10.1186/s12884-018-1937-4) contains supplementary material, which is available to authorized users.

## Background

FGM is the collective name given to several different traditional practices that involve the partial or total removal of the external female genitalia or other injury to the female genital organs for cultural or non-therapeutic reasons [1, 2]. The World Health Organization (WHO) classified FGM into four broad categories: namely Type I or Clitoridectomy, removal of the prepuce with or without excision of the clitoris.; Type II or Excision:, Partial or total removal of the clitoris and the labia minora, with or without excision of the labia majora; type III or Infibulation, excision of labia minora and/or labia majora, with or without excision of the clitoris and stitching of the exposed walls of the labia majora together leaving only a small hole for the passage of urine and menstrual flow; and type IV or unclassified, refers to any other damage to the female genitalia including, pricking, piercing, incising, scraping and cauterization [3]. The practice is carried out by various religions including Christians, Muslims and Ethiopian Jews (Falashas) [4]. Type I and type II are the two most common forms of FGM practiced among Ethiopian women and girls, type II being the most common. Type III, the most drastic form of FGM, is high prevalent among Afar and Somali, but it is also practiced to lesser extent in Harari. Type IV is carried out mainly by the Amhara population.

Even though there are international and local efforts to end FGM, its prevalence rate is still very high in some countries, especially in Africa, Asia and the Middle East [3]. WHO estimated that globally up to 140 million girls and women have been subjected to some type of FGM [3]. In Africa (Sub-Saharan Africa, Egypt and Sudan), about three million women & girls are mutilated every year. Almost half of these women & girls are from Ethiopia and Egypt [5].

In Ethiopia, FGM affects 23.8 million women and girls with the highest adoption being in Afar region (91.6%), Dire Dawa (92.3%) and Somali region (97.3%) [6]. Even though enough success has not been gained so far, the country has made a promising improvement in reducing the practices of FGM. For instance, the prevalence of FGM was reduced from 80% in 2000 to 74% in 2005, demonstrating that the practice is declining gradually [7, 8].

Although FGM is not undertaken with the intention of inflicting harm, its damaging physical, sexual, and psychological effects make it an act of violence against women and children [1, 6, 9]. The fact that FGM counted usually as the most valued and highly orchestrated cultural practice and social norm, women apart from being victimized, they also take part in its perpetration making FGM unique among all other forms of violence with gender base [10]. The ill effects of mutilation is sound occurring at different phases of women’s life when suffering starts as a child at the time of the operation, through their marriage and at the event of child bearing [9]. Traditions as FGM have been regarded as violating of basic human right of women particularly the right to life free from discrimination, ill treatment and torture [3].

FGM is often motivated by a variety of beliefs endorsing it for perceived health and hygiene benefits, traditional, religious requirement and gender related reasons. Although the reasons that drive the practice of FGM widely varies between societies but a common reason given for the practice is that it reduces the sexual desire of girls and to ensure virginity before marriage and fidelity afterward, chastity and the aesthetic appearance of the female body [11, 12]. Most importantly, for many ethnic groups, an uncut girl is considered to be sexually promiscuous and, therefore, unworthy of marriage. The belief also exists that uncut external female genitals are too masculine or unclean. Although FGM is not endorsed by either the Quran or the Bible, supposed religious doctrine is often used by justify the practice [4]. Since mutilating a girl is a kind of tradition with high acceptance and an intensely engrained social, economic and political ground, leaving a girl not to be mutilated is not even an option, facing solid disapproval at a community level [11].

FGM has no health benefits [6]. One of the major problems related to FGM is the difficulty experienced during childbirth [9]. Even though there is no agreed cutoff age during which an FGM is performed, commonly it is done in girls aged 10 years or less, exposing them to varying degree of scar formation [13]. Such scars in turn results in vaginal stenosis, where its wall would fail to progressively be dilated, secondary to the loss of its natural elasticity favoring to the arrest of the decent of the fetal presenting part at birth [14]. Furthermore, such narrowing of the vaginal wall will put both the mother and fetus at risk of morbidity and mortality associated with obstetric complications like prolonged labour, obstructed labour and excessive thinning of the scar due to a pressure excreted by a descending part of the fetus will end up with vaginal and perineal injury [9].

It is very important that this aspect is not ignored when discussing the problem of maternal mortality in Africa [15]. In industrialized countries, maternal deaths owing to childbirth are rare. But in Africa especially in Sub-Saharan Africa [16] including Ethiopia [17] childbirth complications remain the most frequent cause of maternal deaths [18]. The risk of dying during childbirth increases greatly for both the mother and the infant if the mother has been genitally mutilated [15].

The consequences can be even more devastating in resource deprived areas in which only few births attended by qualified health professionals in hospital settings [19]. The high incidence of postpartum haemorrhage, a life-threatening condition, is of particular concern where health services are weak or women cannot easily access them [16]. However, the causal connection of FGM to childbirth related health complication is not commonly known amongst FGM practicing communities. There is thus a clear need for research findings to be communicated not only in international settings but also at national and local level to raise awareness of the serious health consequences connected with it [19]. Therefore, this study is intended to contribute in narrowing the information gap and provide evidence-based information on the effect of FGM on birth complication among mothers who gave birth in Karamara hospital, Jijiga, eastern Ethiopia.

## Methods

### Study area

The study was conducted at Karamara hospital, Jijiga town, eastern Ethiopia between October and December, 2014. Jijiga, capital of the Ethiopian Somali region (ESR), is located at 628 Kms east of Addis Ababa. The town has an elevation of 1609 m above sea level with semi-arid climate. There is one public hospital (Karamara) and one private hospital (Dire), two health centers, one maternal & child health clinic, and around thirty higher and medium clinics. Based on population projection 2007 in to 2013/14 the population of Jijiga town is estimated to be 125,876 from which male accounts for 67,125 while female are 58,748.

### Study design, source population and study participants

A facility based prospective cohort study design was employed. All pregnant women visiting Karamara hospital for delivery services were our source population. Two groups of study participants were involved namely: exposed; those parturients with FGM and unexposed; those parturients who didn’t have FGM. Women were not included if they were appointed for elective caesarean section, if they were arriving in the second stage of labour, if they were not in the gestational age of 37–42 and if they did not consent to participate. Parturients with foetal mal-presentation were also excluded.

### Sample size and sampling technique

The required sample size was calculated by using Epi info Version 7 software. We estimated the proportion of having prolonged 2nd stage labour to be 8.6 and 21.6% among parturients without and with FGM, respectively [20]. With an additional assumption of 95% confidence level, 80% power and 10% non-response rate in our estimate, a total sample of 292 was needed (146 parturients in each group). The study subjects were identified by using consecutive sampling technique. Until the computed sample reached, all women who came to the hospital for delivery and who were volunteer to participate in the study were recruited as study participants.

### Data collection and quality control

Structured interviewer guided questionnaires and observational checklists were employed as data collection tools (Additional file 1). The questionnaire consisted of questions about women’s socio-demographic characteristics and obstetric and medical histories while the observational checklist was used for clinical examination and to collect information on the type of birth complication a women had. The questionnaire was first designed in English based on information from different publications developed for similar purpose [13, 21–23]. Then, the questionnaire was translated to Somali (the local language of the study area) (Additional file 2). Similarly, the checklist was developed by adopting the WHO FGM classification system to identify and categorize women’s status of FGM and the type of FGM she had [24].

Pre-testing of the translated questionnaire was done on 5% of the total sample size in Degehabur hospital, which is located in Degehabur town, 153 Km northwest of Jijiga. It shares similar geographic, economic, cultural and socio-demographic characteristics with Jijiga town. During pre-testing the questionnaire was assessed for its clarity, understandability, length and completeness.

We recruited eight trained, diploma graduated midwives as data collectors and two BSc midwives for supervision, who participated throughout the data collection time. Information concerning the socio-demographic and economic characteristics, reproductive and medical histories of the study participants was collected through the pre-tested interviewer administered questionnaire. An antepartum examination of the external genitalia was performed by the data collectors to determine if the participating women had had FGM or not. Women’s FGM status was classified according to the WHO classification system. The women’s nutritional status was measured by using Mid-Upper Arm Circumference (MUAC) tape. Then, they were followed up until delivery. Details of delivery and data on adverse obstetric outcome were collected at the end of delivery by using the observational checklist. A woman was defined as having a birth complication if it is reported by the birth attendants.

### Study variables

Birth complications such as prolonged 2nd stage of labour, outlet obstruction, perineal tear, postpartum blood loss, emergency caesarean section, appearance pulse grimace activity and respiration (APGAR) score and stillbirth were the main outcome of the study. The independent variables included in the data analysis were FGM, age, religion, ethnicity, income, educational status, occupation, residency, marital status, travel distance, adverse obstetric history, parity, gravidity, antenatal care (ANC) visit, existing illness during admission, height, nutritional status (MUAC), birth weight, plan for pregnancy, life-style/personal habits during pregnancy.

### Data processing and analysis

After data collection, each questionnaire was checked for completeness, inconsistencies, cleaned then coded and entered in to SPSS for windows version 16.0. SPSS version 16.0 and STATA version 11 were used for data analysis. Descriptive statistics like frequency tables, graphs and descriptive summaries were carried out to describe the study variables. The differences in the proportion of the characteristics of women with and without FGM were compared using chi-square test. Bivariate analysis was done to test the association between the independent and the outcome variables. A *P*-value of < 0.25 was used to select candidates for the multivariate analysis. Then, to control the confounding effect of other variables and to determine independent predictors of birth complication, multivariable log-binomial regression analysis was carried out by taking significant variables in the bivariate analysis. The risks of independent variables on adverse birth outcome were assessed using the risk ratio as the measure of effect. Estimates, together with corresponding 95% confidence interval of the estimates were computed. Statistical significance was declared at *P* < 0.05.

## Results

### Socio-demographic characteristics

**Fig. 1:**
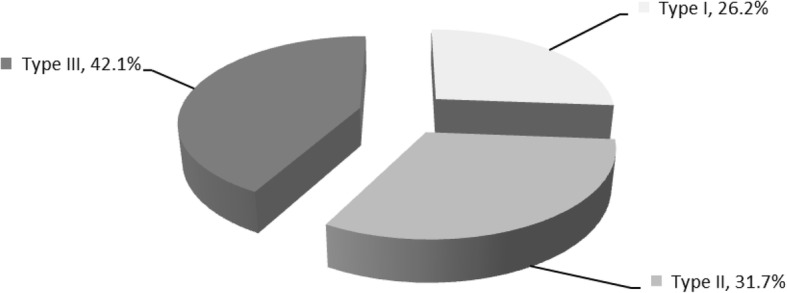
Proportion of genital cutting seen among exposed parturients in Karamara hospital, Jijiga, eastern Ethiopia, from October to December, 2014

The study sample consisted of 281 parturients, of which 142 with FGM and 139 without FGM (Table 1). The mean age was 25.4 years (SD 5.69) and 26.1 years (SD 4.86) among parturients with and without FGM respectively. A significantly high proportion of Muslim (88.7% versus 59%, *P* < 0.001) and Somali (72.5% versus 40.3%, p = < 0.001) women were found in the exposed group, compared to the non-exposed group. The majority of the study participants were housewife, had no formal education and were urban residents in both groups. Women of the Somali ethnic group had a higher frequency of FGM (72.5%) followed by those of the Oromo (13.4%) and Amara (9.2%). Among women with FGM, 26.2, 31.7, and 42.1% had type I, type II and type III FGM respectively (Fig. 1).

**Table 1:** Socio-demographic and economic characteristics of parturients in Karamara hospital, Jijiga, eastern Ethiopia, from October to December, 2014

Background characteristics	Non-exposed (n-139)	Exposed (n-142)	*P*-value
	Number (%)	Number (%)	
Age (years)			
< 20	18(12.9)	37(26.1)	0.048
20–24	36 (25.9)	28(19.7)	
25–29	53(38.1)	48(33.80)	
≥ 30	32(23.0)	29(20.4)	
Mean (SD)	26.15(4.86)	25.40(5.69)	
Religion			
Muslim	82(59)	126(88.7)	< 0.001
Orthodox	53(38.1)	13 (9.2)	
Protestant	4(2.9)	3(2.1)	
Ethnicity			
Somali	56 (40.3)	103(72.5)	< 0.001
Amhara	41 (29.5)	13(9.2)	
Oromo	31(22.3)	19(13.4)	
Others	11(7.9)	7(4.9)	
Educational status			
No formal education	98(70.5)	105(73.9)	0.074
primary	20(14.4)	17(12)	
Secondary & above	21(15.1)	20(14.1)	
Occupation			
Housewife	87(62.6)	83(58.9)	0.788
Student	8(5.8)	12(8.5)	
Daily Labour	6(4.3)	10(7.1)	
Merchant	14(10.1)	18(12.8)	
Government employee	12(8.6)	11(7.8)	
Private employee	12(8.6)	7(5)	
Income			
< 500	21(15.4)	23(16.2)	0.507
500–999	42(30.9)	33(23.2)	
1000–1499	29(21.3)	31(21.8)	
≥ 1500	44(32.4)	55(38.7)	
Residence			
Urban	120(87.6)	121(85.2)	0.343
Rural	17(12.4)	21(14.8)	
Height (cm)			
≤ 150	7(5)	13(9.2)	0.063
> 150	132(95)	128(90.8)

### Past obstetric history of the study participants

There was a statistically significant difference in the distribution of parity between the two groups (p = < 0.001) (Table 2). Nulliparous was observed more in the exposed (52.8%) than in non-exposed (30.2%). The highest proportion (62.6%) in the non-exposed groups had parity of 1–3 compared to the exposed group (39.4%). With regards to the distribution of respondents’ gravidity, the mutilated mothers had a higher mean gravidity compared to the non-mutilated (1.91 ± 0.71 versus 1.71 ± 0.82). In the non-exposed group, primigravid had the largest proportion of the study participants (47.5%), whereas in the exposed group, multigravida had the largest proportion (66.2%). A slightly higher proportion of women in the exposed group (9.2%) had a history of stillbirth than that of non-exposed (5%). There was however, no significant difference in both groups in terms of the respondents history of abortion (*P* = 0.133) and stillbirth (*P* = 0.505).

**Table 2:** Past obstetric history of parturients in Karamara hospital, Jijiga, eastern Ethiopia, from October to December, 2014

Variables	Unexposed (*N* = 139)	Exposed(*N* = 142)	*P*-value
	Number (%)	Number (%)	
Gravidity			
Primigravid	37(26.6)	67(47.5)	0.002
2–4	92(66.2)	61(43.3)	
≥ 5	10(7.2)	13(9.2)	
Mean (SD)	1.91(0.71)	1.71(0.82)	
Parity			
Nulliparous	42(30.2)	75(52.8)	< 0.001
1–3	87(62.6)	56(39.4)	
≥ 4	10(7.2)	11(7.7)	
Mean (SD)	1.31(1.35)	1.07(1.70)	
History of abortion			
Yes	6(4.3)	7(5)	0.133
No	133(95.7)	133(95)	
History of still birth			
Yes	7(5)	13(9.2)	0.505
No	132(95)	129(90.8)	

### History of current pregnancy

With regards to reproductive health characteristics, there was a significant difference between the two groups of mothers, with a majority (53.5%) of the exposed group not obtaining ANC services compared with the non-exposed (30.2%) P = < 0.001 (Table 3). However, there was no significant difference in the plan for pregnancy, illness during admission, MUAC and distance traveled to reach to the hospital.

**Table 3:** History of current pregnancy of parturients in Karamara hospital, Jijiga, Eastern Ethiopia, from October to December, 2014

Variables	Non-exposed (N = 139)	Exposed (N = 142)	*P*-value
	Number (%)	Number (%)	
Plan for Pregnancy			
Yes	111(79.9)	117(82.4)	0.348
No	28(20.1)	25(17.6)	
Number of ANC visits			
None	42(30.2)	76(53.5)	< 0.001
1–3	85(61.2)	56(38.7)	
≥ 4	12(8.6)	11(7.7)	
Illness during admission			
Yes	4(2.9)	5(3.5)	0.515
No	135(97.1)	137(96.5)	
MUAC (cm)			
< 21	45(32.6)	44(31)	0.435
≥ 21	93(67.4)	98(69)	
Distance traveled (Mints)			
≤ 60	111(81)	116(81.7)	0.504
> 60	26(19)	26(18.3)	

### Frequency of birth complications

**Fig. 2:**
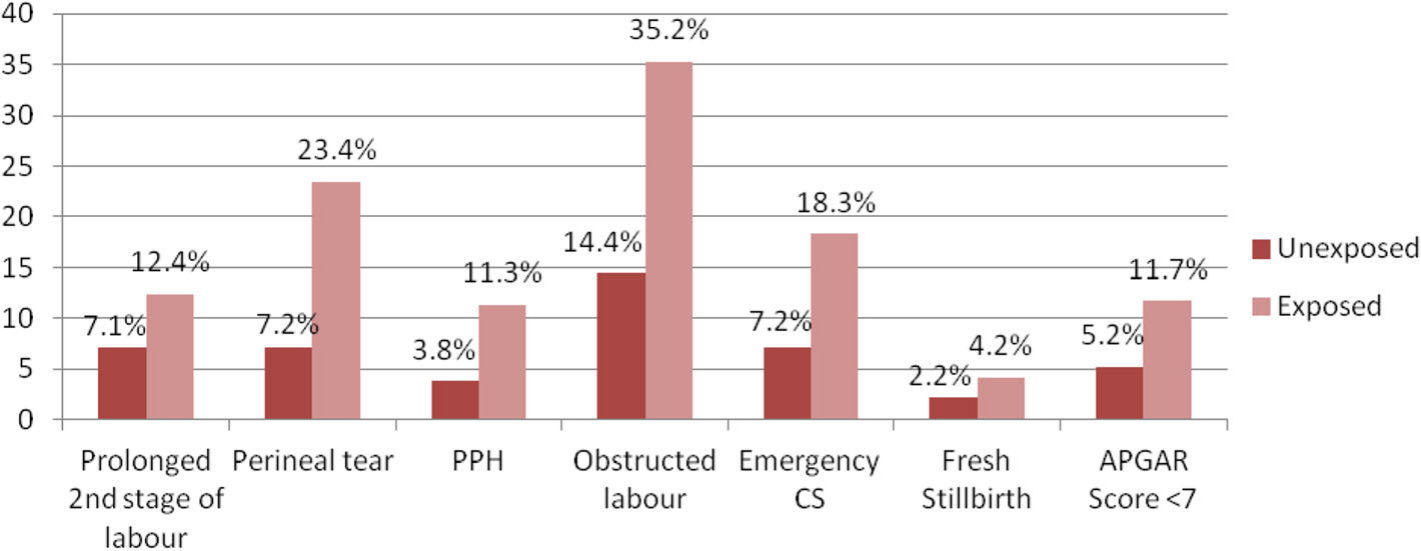
Proportion of birth complication among parturients with and without FGM in Karamara hospital, Jijiga, eastern Ethiopia, from October to December, 2014

The findings of this study implicated that women with FGM were more likely than women without FGM to experience obstructed labour, perineal tear and postpartum blood loss (Fig. 2). At the same time, the frequency of prolonged 2nd stage of labour was slightly higher in the exposed (12.4%) than in the non-exposed group (7.1%).

### FGM and its association with birth complications

The multivariate analysis reveals that the risk of prolonged 2nd stage of labour in the exposed group was 1.33 times higher than the unexposed; however the association was not statistically significant (Table 4). Regarding perineal tear, we found a significant effect favoring the non-exposed group [RR 2.52 (95% CI, 1.26–5.02)]. Similarly, the risk of having postpartum blood loss was 3.14 times higher among women with FGM than their counterpart. The risk of outlet obstruction was also observed to be significantly associated with FGM status of the parturients. In this regard, the exposed parturients had 1.83 times higher risk of obstructed labour than the non-exposed groups [RR = 1.83 (95% CI 1.19–2.79)]. Women with FGM had a 2.31 times higher risk of emergency caesarean section than those without FGM and the observed difference was statistically significant. Infants born to women with FGM were found to have a 1.35 times higher risk of having APGAR score < 7 than infants from unexposed women; however the observed difference was not statistically significant.

**Table 4:** The association of FGM with birth complication among parturients with (*n* = 142) and without FGM (*n* = 139) in Karamara hospital, Jijiga, eastern Ethiopia, from October to December, 2014

Types of complication	Complication		CRR(95%CI)	*P*-value	ARR (95% CI)^a^
	No	Yes			
	Number (%)	Number (%)			
Prolonged 2nd stage of labour					
Non-exposed	118 (92.9)	9(7.1)	1		1
Exposed	120(87.6)	17(12.4)	1.75(0.80–3.75)	0.154	1.33(0.56–3.16)
Perineal tear					
Non-exposed	128(92.8)	10(7.2)	1		1
Exposed	108(76.6)	33(23.4)	3.22(1.65–6.29)	0.001	2.52(1.26–5.02)*
Postpartum blood loss					
Non-exposed	134(96.4)	5(3.6)	1		1
Exposed	126(88.7)	16(11.3)	3.13(1.17–8.31)	0.022	3.14(1.27–7.78)*
Outlet obstruction					
Non-exposed	119(85.6)	20(14.4)	1		1
Exposed	92(64.8)	50(35.2)	2.44(1.54–3.88)	< 0.001	1.83(1.19–2.79)*
Emergency CS					
Non-exposed	129(92.8)	10(7.2)	1		1
Exposed	116(81.7)	26(18.3)	2.54(1.27–5.07)	0.008	2.31(1.10–4.82)*
APGAR score < 7					
Non-exposed	127(94.8)	7(5.2)	1		1
Exposed	113(87.6)	16(12.4)	2.37(1.01–5.58)	0.047	1.35 (0.56–3.2)
Fresh stillbirth^b^					
Non-exposed	136(97.8)	3(2.2)			
Exposed	136(95.8)	6(4.2)			

^a^ Adjusted for potential confounders (parity, age, birth weight, educational status, ANC visit, height, distance traveled and MUAC) * *P* < 0.05 ^b^Not calculated because of insufficient number of cases

### The risk of birth complication in relation to the type of FGM

The result of this study revealed that the risk of obstetric complications increased with the severity of the procedure (Table 5). Women with type II and III FGM were significantly more likely to suffer from childbirth related complications than parturients with type I. Women with type III FGM were significantly more likely to have a caesarean section and postpartum blood loss of 500 ml or more than women who had not had FGM. The relative risk (RR) of having emergency caesarean section was 2.00 (95% CI 0.74–5.85)] for women with type I FGM, 2.40 (95% CI 0.94–6.08)] for those with type II, and 3.60 (95% CI 1.65–7.86)] for those with type III, compared to women with no FGM. When women with caesarean section were excluded from the analysis of postpartum haemorrhage, the RRs were 0.93 (95% CI 0.10–8.15) for type I FGM, 2.31 (95% CI 0.53–9.96) for type II, and 6.37 (95% CI 2.11–19.20) for type III, compared to women without FGM. The risk of having perineal tear was significantly increased in women who had under gone type II [RR = 2.45 (95% CI 1.03–5.83)] and III FGM [RR = 4.91(95% CI, 2.46–9.77)] than women without FGM. Similarly, parturients with type II and type III FGM were found to be 2.38 and 2.94 times more likely to be complicated by outlet obstruction respectively, than women without FGM. On the other hand, parturients with type I FGM were found to have 0.68 times less likely to have outlet obstruction than women without FGM, even though the association was not statistically significant [RR = 0.68 (95% CI 0.25–1.85)]. Infants born to women with type I and type II FGM were 0.54 and 0.95 times less at risk of having APGAR score of < 7 respectively, compared to infants of unexposed women, without significant statistical difference. Significantly, infants born to women with type III FGM had a 4.41 times higher risk of having an APGAR score of < 7 compared to infants of unexposed women.

**Table 5:** Difference in risk of adverse obstetric outcomes in women with FGM I, II, & III compared with women without FGM in Karamara hospital, Jijiga, eastern Ethiopia, from October to December, 2014

Type of birth Complication	Number (%)	RR (95% CI)	*P* value
Prolonged 2nd stage of labour			
No FGM	9(7.1)	1	
FGM I	2(5.6)	0.78(0.17–3.46)	0.748
FGM II	5(11.4)	1.60(0.56–4.52)	0.373
FGM III	10(17.5)	2.47(1.06–5.76)	0.035
Emergency Caesarean Section			
No FGM	10(7.2)	1	
FGM I	5(13.5)	2.00(0.74–5.85)	0.162
FGM II	7(15.6)	2.40(0.94–6.08)	0.064
FGM III	14(23.3)	3.60(1.65–7.86)	0.001
Perineal tear			
No FGM	10(7.2)	1	
FGM I	4(10.8)	1.49(0.49–4.48)	0.476
FGM II	8(17.8)	2.45(1.03–5.83)	0.042
FGM III	21(35.6)	4.91(2.46–9.77)	< 0.001
Outlet obstruction			
No FGM	20(14.4)	1	
FGM I	6(16.2)	0.68(0.25–1.85)	0.456
FGM II	16(35.6)	2.38(1.39–4.08)	0.001
FGM III	38(46.7)	2.94(1.84–4.71)	< 0.001
Postpartum blood loss			
No FGM	4(2.9)	1	
FGM I	1(2.7)	0.93(0.10–8.15)	0.955
FGM II	3(6.7)	2.31(0.53–9.96)	0.205
FGM III	11(18.3)	6.37(2.11–19.20)	0.001
APGAR score **< 7**			
No FGM	7(31.8)	1	
FGM I	1(4.5)	0.54(0.06–4.29)	0.548
FGM II	2(9.1)	0.95 (0.20–4.42)	0.955
FGM III	12(19.9)	4.41(1.84–10.60)	0.001

## Discussion

The current study attempted to determine the association of FGM with birth complications. Deliveries to women with FGM are more likely to be complicated by perineal tear, postpartum hemorrhage, emergency caesarean section and obstructed labour, compared to deliveries to women without FGM. However, the association between FGM and the risk of having an infant with low-APGAR score and prolonged second stage of labour was not statistically significant.

In this study, a clear positive relationship between the type of FGM and the likelihood that a women will have a visible obstetric complications, as the severity of the cut increases the risk of having complication will also increases. All types of complications are more prevalent among women with type II and type III cuts than among those with a type 1 cut. This finding was also reported in different studies [13, 20, 22, 25, 26].

One of the birth complications most often found among women with FGM was obstructed labour, with a frequency of 2.5 times higher for women with type II FGM (35.6%) and 3.0 times higher for women with type III FGM (46.7%) compared to women who had not undergone FGM (14.4%). The observed increased risk of obstructed labour among women with FGM is consistent with other studies [13, 22, 27]. The higher frequency of obstructed labour may be attributed to loss of elasticity of the perineal tissue. This is because the scar (fibrosis and keloids), which are formed after excision increases the fragility of the tissues [28]. The use of episiotomy or expediting delivery using instrument might be acceptable to be carried out with a prime objective of preventing the risk of trauma on the genital tract especially in women with type II & III mutilations. In fact, episiotomy is often needed for women with infibulations, particularly for first deliveries [29].

Similarly, greater chance of developing perineal tear at the time of birth has been significantly associated with type II and III FGM. Our findings persisted even after adjusting for major risk factors. This can be well explained by the scarring of the introitus following FGM, which reduces its elasticity. The perineum being unscarred, gives way easily during the second stage of labour because of overstretching. Moreover, the scarred part of the introitus is tougher than the perineum. The increased risk of perineal tear among exposed women in this study is similar to findings from earlier studies [20, 22, 30].

Of more relevance is the finding that FGM was statistically associated with postpartum blood loss. Parturients with FGM had 3.14 times higher risk of postpartum blood loss than Parturients without FGM. The associations between FGM type I and FGM type II was however not significant but FGM type III with RR of 6.37 (95% CI 2.11–19.20) was strongly associated with postpartum blood loss. This finding is also consistent with other studies [13, 22, 31]. This might be explained by the fact that one of the Long-term complications of FGM is formation of scars and keloid on the perineal and vaginal tissues. This scar tissue is less elastic and not as strong as healthy perineal and vaginal tissues. There for the presence of this scar tissue, might be attributed to increase bleeding from high rate of laceration and tears or episiotomy. Therefore, this could underlie the findings of an increased risk of postpartum blood loss.

Furthermore, this study revealed a significant positive relationship between the practice of FGM and emergency cesarean section. Parturients who were exposed to the practice had 2.31 times higher risk of delivery by emergency cesarean section than non-exposed parturients. In addition, our study also revealed that the risk of having emergency cesarean section increase with the severity of the procurers in which parturients with FGM type III had significantly higher risk of emergency cesarean section. Finding is consistent with previous studies [21, 22, 27, 31]. This is because when the vagina is seen to be very rigid, it may leads to severe vaginal lacerations or third degree perianal tears. Therefore, it is likely that caesarian section will be the best option to manage the labor. In addition, if keloids have formed and are too large, a caesarian section might be the best option to deliver [14].

In the current study, women with and without FGM did not have significantly different risks of prolonged 2nd stage of labour, which is similar to a study conducted in Switzerland [30]. However, a study conducted in Sweden [32] revealed that parturients with FGM had a significantly shorter 2nd stage of labour. It is likely that scarring from FGM would be too delicate to be torn during delivery and these tears may be related to scar tissue with decreased tensile strength. Scar tissue consists of highest concentration of mature collagen after recurrent incision and healing, indicating the importance of inflammatory activity [33]. Thus, porous women after FGM may have a reduction in tissue strength and, therefore, a greater probability of vaginal tears.

This study demonstrated no association between FGM and low APGAR score. The difference in the proportion of poor foetal outcome (low APGAR score) among both groups was not statistically significantly. Our finding is different from a case control study conducted in San Camillo Hospital, Burkina Faso in which low APGAR score was significantly higher among case than control group [27]. The difference can be explained due to differences of study design.

### Limitation

The data presented here were primarily collected through direct observation by trained medical staff, and they appear to be of greater validity than women’s self-reports. However, the study has potential limitation. Since there was no post delivery follow-up, late complications like fistula and maternal death were not evaluated.

## Conclusion

The study showed that women with FGM are significantly more likely than those without FGM to have adverse obstetric outcomes such as outlet obstruction, perineal tear, postpartum blood loss and emergency caesarean section. Risks seem to be greater with more extensive forms of FGM. This means that FGM is likely to be responsible for substantial numbers of additional causes of adverse obstetric outcomes. Adverse obstetric outcomes can therefore be added to the known harmful immediate and long-term effects of FGM.

## Additional files

Additional file 1: Questionnaires administered in the study.doc, 51.5 K. The questionnaire has all the questions that were used to collect data reported within the manuscript. (DOCX 45 kb)

Additional file 2: Somali version of the questionnaire for the study of the sequela of FGM on birth outcomes in Jijiga town, Ethiopian Somali region, October to December, 2014. (DOC 137 kb)

## Abbreviations

ANC: Antenatal care
APGAR: Appearance Pulse Grimace Activity and Respiration
CI: Confidence interval
ESR: Ethiopian Somali region
FGM: Female genital mutilation
MUAC: Mid-upper arm circumference
RR: Relative risk
SD: Standard deviation
SPSS: Statistical package for social science
WHO: World health organization

## References

[1] Center for Reproductive Rights (2006). Female genital multilation,a matter of human rights:an advocate’s guide to action. 2^nd^ edition.

[2] World Health Organization. Female genital mutilation: report of a technical working group. Geneva: World Health Organization; 1995.

[3] World Health Organization (2008). Eliminating female genital mutilation: an interagency statement.

[4] United States Department of State, Ethiopia: Report on Female Genital Mutilation (FGM) or Female Genital Cutting (FGC), 1 June 2001, available at: http://www.refworld.org/docid/46d57877c.html.

[5] United Nations Children’s Fund Innocent Research Center (2005). Changing a harmful social convention: female genital mutilation/cutting.

[6] Allen K, Marshall D, Morris C, Waritay J, Wilson AM (2013). FGM in Ethiopia: country profile. 28 too many.

[7] Central Statistical Authority [Ethiopia] and ORC Macro (2001). The 2000 Ethiopia demographic and health survey.

[8] Central Statistical Agency [Ethiopia] and ORC Macro (2006). Ethiopia Demographic and Health.Survey 2005.

[9] African women organization. The consequences of female genital mutilation: *African- women ORG*; 2009. [http://www.african-women.org/FGM/consequences.php]

[10] World Health Organization. Understanding and addressing violence against women. Geneva; 2012. [http://www.who.int/reproductivehealth/publications/violence/en/index.html]

[11] END FGM–European campaign. Ending female genital mutilation, a strategy for the European Union institutions: an executive summary. Brussels: END FGM – European Campaign. [http://www.endfgm.eu/content/assets/END_FGM_Final_Strategy_Summary.pdf]

[12] Dorkenoo E, Morison L, Macfarlane A (2007). A statistical study to estimate the prevalence of female genital mutilation in England and Wales: a summary report. Foundation for Women’s Health, Research and Development.

[13] Banks E, Meirik O, Farley T, Akande O, Bathija H, Ali M (2006). Female genital mutilation and obstetric outcome: WHO collaborative prospective study in six African countries. Lancet.

[14] Ismail EA. Female genital mutilation survey in Somaliland at the Edna Adan maternity and teaching hospital. Hargeisa: Edna Adan Maternity Hospital; 2009.

[15] Desert Flower (2010). FGM major factor in high maternal mortality rates in Africa.

[16] World Health Organization. Maternal mortality, WHO Media centre: Fact sheet No.348. Geneva; 2012. [http://www.who.int/mediacentre/factsheets/fs348/en/]

[17] Abdella A (2010). Maternal mortality trend in Ethiopia. Ethiop J Health Dev.

[18] United Nations Population Fund:No women should die giving life: facts and figures. The Lancet’s Maternal Survival and Women Deliver Series 2006/2007. [http://www.unfpa.org/safemotherhood].

[19] Amnesty International. End FGM: ending female genital mutilation to promote the achievement of the millennium development goals. Brussels: Amnesty International; END FGM – European Campaign; 2010.

[20] Kaplan A, Forbes M, Bonhoure I, Utzet M, Martín M, Manneh M, Haruna Ceesay H (2013). Female genital mutilation/cutting in the Gambia: long-term health consequences and complications during delivery and for the newborn. Int J Women’s Health.

[21] Oduro AR, Ansah P, Hodgson A, Afful AM, Baiden F, Adongo P, Koram KA (2006). Trends in the prevalence of female genital mutilation and its effect on delivery outcomes in the Kassena-Nankana District of northern Ghana. Ghana Med J.

[22] Ndiaye P, Diongue M, Faye A, Ouedraogo D, Tal-Dia A (2010). Female genital mutilation and complications in childbirth in the province of Gourma. Burkina Faso.

[23] Ravi RP (2013). Does socio-demographic determinants influence complications while delivery among young married women in TamilNadu, India. IJMCH.

[24] World Health Organization. Classification of female genital mutilation. Geneva: World Health Organization; 2008.

[25] Jones H, Diop N, Askew I, Kabore I (1999). Female genital cutting practices in Burkina Faso and Mali and their negative health outcomes. Stud Fam Plan.

[26] Kwasi O-A (2005). Female genital mutilation and obstetric squeal in the upper east region of Ghana.

[27] Frega A, Puzio G, Maniglio P, Catalano A, Milazzo GN, Lombardi D, Nitiema H, Bianchi P (2013). Obstetric and neonatal outcomes of women with FGM I and II in san Camillo hospital, Burkina Faso. Arch Gynecol Obstet.

[28] Rushwan H (2000). Female genital mutilation (FGM) management during pregnancy, childbirth and the postpartum period. Int J Gynecol Obstet.

[29] Virginia L, Marcotte K. Obstetric complications of Somali female circumcision. Obstet Gynecol. 1999;93(4):2-19.

[30] Wuest S, Raio L, Wyssmueller D, Mueller MD, Stadlmayr W, Surbek DV, Kuhn A (2009). Effects of female genital mutilation on birth outcomes in Switzerland. BJOG.

[31] Chibber R, El-Saleh E, El Harmi J (2011). Female circumcision: obstetrical and psychological sequelae continues unabated in the 21st century. J Matern Fetal Neonatal Med.

[32] Essen B, NO S¨B, Gudmundsson S, Ö stergren PO, Lindquist PG (2005). No association between female circumcision and prolonged labour: a case control study of immigrant women giving birth in Sweden. Eur J Obstet Gynecol Reprod Biol.

[33] Souza ZA, Greca FH, Noronha L, Maranha AS, Calil AP, Hubie DP, Barbosa FM (2007). Abdominal wall healing in repeated rats. Acta CirBras.

