# Supplementary material for: Sequela of female genital mutilation on birth outcomes in Jijiga town, Ethiopian Somali region: a prospective cohort study
Source: BMC Pregnancy Childbirth. 2018 Jul 20;18:305. doi: 10.1186/s12884-018-1937-4 (PMC6053719; doi:10.1186/s12884-018-1937-4)
Supplement: Supplementary file 1 — Questionnaires administered in the study.doc, 51.5 K. The questionnaire has all the questions that were used to collect data reported within the manuscript. (DOCX 45 kb) [file 12884_2018_1937_MOESM1_ESM.docx]

**Questionnaire for study on female genital mutilation and birth complications among women’s who come to health facility for delivery services, Jig-jiga town, Somali region, eastern Ethiopia, from October to December, 2016.**

Date: ___ / ___ / ___

**Section 1:- FGM related information (based on observation)**

Notice: Use the following description to ascertain the type of FGM

| **S No** | **Question** | **Observation** | **Skip to** |
| --- | --- | --- | --- |
| Q101 | Does the woman have any type of FGM? **Observe and record!** | 1. Yes 2. No | Go to Q201 |
| Q102 | If yes what type of FGM?  **Observe and record!** | 1. Type I   Excision of partial or total of the clitoris and/or the clitoral hood   1. Type II   Partial or total removal of the clitoris and the labia minora, with or without excision of the labia majora.   1. Type III   The removal of labia minora and/or labia majora, with or without excision of the clitoris and stitching and/or narrowing of the vaginal opening leaving a small hole for urine and menstrual flow.   1. Type IV   All other harmful procedures to the female genitalia for non-medical purposes (e.g., pricking, piercing, incising, scraping, and cauterization. |  |

**Section 2:- Respondent background information**

| **S.No** | **Question** | **Coding categories** | **Skip to** |
| --- | --- | --- | --- |
| Q201 | How old are you? (Age in years) | ___________ Years |  |
| Q202 | Where is your residence? |  |  |
| Q203 | What is the highest level of School you completed? | 1. Unable to read & write 2. Able to read & write   Highest grade completed ___________ |  |
| Q204 | What is your religion | 1. Muslim 2. Orthodox 3. Protestant 4. Catholic 5. Others(Specify)___________ |  |
| Q205 | What is your ethnicity? | 1. Somali 2. Amhara 3. Oromo 4. Tigre 5. Other (Specify)__________ |  |
| Q206 | What is your marital status? | 1. Single/never married 2. Married/Living together 3. Divorced/separation 4. Widowed |  |
| Q207 | What do you do for living?  {Or What is your occupation | 1. Housewife 2. Student 3. Daily laborer 4. Merchant 5. Government Employee 6. Private employee 7. Other (specify) ____________ |  |
| Q208 | How much is the average family income per month? | ________________ Birr   - - - 1. No income   88. Don’t now |  |
| Q209 | How many minutes’ walk is it from your residence to the hospital? | ___________ No of minutes |  |

**Section 3: Past obstetric history**

| **S.No** | **Question** | **Coding categories** | **Skip to** |
| --- | --- | --- | --- |
| Q301 | Have you had any pregnancy before? | 1. Yes 2. No | Go to Q401 |
| Q302 | How many pregnancies have you had? | Number of pregnancies ___________ |  |
| Q303 | How many times did you give birth? (Parity) | Number of times______________ |  |
| Q304 | Have you ever-experienced still birth? (Birth of dead fetus after 28 weeks of gestation) | Yes  No | Go to Q307 |
| Q305 | How many times did you have still births? | Number of still births ____________ |  |
| Q306 | Have you ever-experienced miscarriage/ abortion? (That is any pregnancy terminated before 28 weeks of gestation) | Yes  No | Go to Q401 |
| Q307 | How many abortions did you have? | Number of abortions_____________ |  |

**Section 4:- Current pregnancy history**

| **S.No** | **Question** | **Coding categories** | **Skip to** |  |
| --- | --- | --- | --- | --- |
| Q401 | Have you planned this Pregnancy? | 1. Yes 2. No |  |  |
| Q402 | Did you see any health professional for antenatal care for this pregnancy? | 1. Yes 2. No | Go to Q404 |  |
| Q403 | How many times in total did you go for antenatal care during this pregnancy? | No of visit______ |  |  |
| Q404 | During this pregnancy have you been told that you have hypertension? | 1. Yes 2. No |  |  |
| Q405 | Have you ever been told that you have DM? | 1. Yes 2. No |  |  |
| Q406 | Did you have any history of trauma or injury, in the current pregnancy period? Probe for all, car accidents etc. | 1. Yes 2. No   88. Don’t remember |  |  |
| Q407 | What is the height of the mother? | ______Height in Cm |  |  |
| Q408 | Mid-upper arm circumference (MUAC) **(Please measure and recorded )** | ______________Cm |  |  |
| Q409 | During your pregnancy, did you take alcohol drinks? | 1. Yes 2. No   88. Don’t remember | Go to Q411 |  |
| Q410 | How often were you taking alcohol drinks?    One unit of alcohol means  1, One glass of beer, Wine, tella, tej, bordi, or  2, one cup of arekie, gin, whisky etc. | 1. Daily 2. 5-6 times per week 3. 3-4 times per week 4. 1 times per week 5. Once a month 6. 3-8 times during pregnancy 7. 1-2 times during pregnancy 8. Don’t remember |  |  |
| Q411 | During your pregnancy, did you ever chew khat? | 1. Yes 2. No   88. Don’t remember | Go to Q413 |  |
| Q412 | How often were you chewing khat? | 1. Daily 2. 5-6 times per week 3. 3-4 times per week 4. 1 times per week 5. Once a month 6. 3-8 times during pregnancy 7. 1-2 times during pregnancy   88. Don’t remember |  |  |
| Q413 | During your pregnancy, did you ever smoke? | 1. Yes 2. No   88. Don’t remember | End  of interview |  |
| Q414 | How often were you smoking? | 1. Daily 2. 5-6 times per week 3. 3-4 times per week 4. 1 times per week 5. Once a month 6. 3-8 times during pregnancy 7. 1-2 times during pregnancy   88. Don’t remember |  |  |

**Section 5:- Information on delivery outcome (Observational)**

**Notes:** This observational checklist should be filled at the end of delivery

| **S No** | **Question** | **Observation** | **Skip to** |
| --- | --- | --- | --- |
| Q501 | What was the mode of delivery | 1. Spontaneous vaginal delivery 2. Spontaneous vaginal delivery assisted by episiotomy 3. Instrumental delivery 4. Cesarean section | Q504 |
| Q502 | What was duration of second stage of labour? | Duration in mints_______ |  |
| Q503 | Does she have perineal tear? | 1. Yes 2. No |  |
| Q504 | Is there Postpartum blood loss? | 1. Yes 2. No | Go to Q506 |
| Q505 | Amount of blood lose | _______estimated volume in ML |  |
| Q506 | Is the labour obstructed? | 1. Yes 2. No |  |
| Q507 | Is the child born alive? | 1. Yes 2. No |  |
| Q508 | Birth weight of the baby in grams?  **Please review and fill from records.** | Weigh in gram ____________________ |  |

***Thank you for your participation!!!***

INTERVIEWER:

Name _______________________________signature _____________Date [___/___/___]

CHECKED BY SUPERVISOR:

Name _______________________________Signature ____________ Date [___/___/___]
