# Supplementary material for: Sequela of female genital mutilation on birth outcomes in Jijiga town, Ethiopian Somali region: a prospective cohort study
Source: BMC Pregnancy Childbirth. 2018 Jul 20;18:305. doi: 10.1186/s12884-018-1937-4 (PMC6053719; doi:10.1186/s12884-018-1937-4)
Supplement: Supplementary file 2 — Somali version of the questionnaire for the study of the sequela of FGM on birth outcomes in Jijiga town, Ethiopian Somali region, October to December, 2014. (DOC 137 kb) [file 12884_2018_1937_MOESM2_ESM.doc]

**SOMALI VERSION QUESTIONNAIRE**

**Tilmaamo guud**

1. Waxaa muhiim ah inaad raacidid talaboyinka wakhtigaad su;aalyso dhamaan su’allaha leh jawaabaha xadidan kadib qor jawaabtooda

- U su’aal sida kuqaran su’aasha
- Ka Dooro jawaabta ugu dhaw
- Ha u akhrin jawaabaha qoran kahor.

**Qaybta 1:-Xog laxidhhaha Fircooniga(Arag ku xaqiijin )**

**FG; Fadlan raac tilmaamahan si aad uxaqiiso nooca firicooniga**

| Tir | Su’asha | fiirin kadib: Malaha Fircooni | Ugudub |
| --- | --- | --- | --- |
| S101 | Gudniinka fircooniga malagugusameeyay Fiiri kadibna diwaangali | - - - 1. Haa  1. Maya | ugudu,S201 |
| S102 | Hadii ay jirto waa noocee Fircoongasi?fiiri kadibna diwaangali | 1. Jarida qayb kamids ah ama dhamaan xubinta dareenka ama 2. Jarida qayb ama dhamaan xubinta dareenka,iayadoo laracinayo qaybta hore ee saxaaxa,tasooy lasocoto tolida qaybta hore . 3. Soo uriir kusamayn daloolak saxaaxa iyadoo toliin lagu samaynayo tasooy lasocon karto jarida xubinta dareenka. |  |

**Qaybta2:- War bixin kusaabasan shaqsiga**

| Tir | **Su;aasha** | **Summada** | **ka gudub** |
| --- | --- | --- | --- |
| S201 | Da’adu waa imisa? (TI) | ___________ SanadkaYears |  |
| S202 | Waxagee deegankagu? | - - - 1. Magallo       2. Miyiga Rural |  |
| S203 | Waxbarshadu halkeed ka gaadhay? | 1, Waxaba aan qorin iyo akhrinakhrinaba  2, Wax qori kara akhrina kara  Fasalkad gaadhay ___________ |  |
| S204 | Diinteed haystaa? | - - - 1. Muslim       2. Ortodhogos       3. Borotestan       4. Kaatolik       5. Tukale(Cadee)___________ |  |
| S205 | Qoomiyadeed tahay? | - - - 1. Soomali       2. Axmaar       3. Oromo       4. Tigrey       5. kale(Cadee)__________ |  |
| S206 | Ma guursatay? | - - - 1. Wali aan guursan       2. Guursaday/Reer leh       3. Carmal ah       4. Kalatgay       5. Dumaal |  |
| S207 | Maxaad ka qashaqaystaa? | - - - 1. Marwo       2. Ardayad       3. Xoogsato       4. Ganacsto       5. Shaqaale dawladeed       6. Shaqale aan dawli ahyn       7. Wax kale (Halkan ku qor) _____ |  |
| S208 | Waa intee dakhliga qooyskiina bishaba? | ________________ Birr  1, Wax kharsha an helinin  88. Ma garankaro |  |
| S209 | Imisa daqiiqo ayay ujirtaa xaafdaadu cuspitalka? | ___________Tirada daqiiqadaha |  |

**Qaybta 3: Xogta Taranka Hooyada ee wakhti hore**

| **Tir** | **Su;aasha** | **Summada** | **Ugudub** |
| --- | --- | --- | --- |
| S301 | Hada ka hor uur ma yeelatey? | 1. Haa 2. Maya | **ugudu,S303** |
| S302 | Ilaa hada Imisa jeer ayaad Uur qaaday/Yeelatay? | Tirada Uur ka______ |  |
| S303 | Imisa jeer ayaadse umashay? | Tirada ______________ |  |
| S304 | Imisa jirr ayaad ahayd marki kugu horaysay eed Umosho? | Da’da oo sanad ________ |  |
| S305 | Waligaa ma umashay ilmo aan shinkiisa(waqtigiisu) gaadhin? | - - - 1. Haa       2. Maya | **ugudu,S307** |
| S306 | Imasa jeer ayaad umashay ilmo aan shinkiisa(waqtigiisa) gaarin | Tirada _____________ |  |
| S307 | Waligaa maad umashay ilmo dhicis ah? | - - - 1. Haa       2. Maya | **ugudu,S401** |
| S308 | Imisa jeer ayaad umashay ilmo dhicis ah? | Tirada_____________ |  |

**Qaybta 4:- Xogta Taranka Hooyada ee Hada**

| Tir | **Su’aasha** | **Summada** | **Ugudub** |  |
| --- | --- | --- | --- | --- |
| S401 | Uurkan ma mid aad qorshaystay baa? | - - - 1. Haa       2. Maya |  |  |
| S402 | Daryeelka Xanaanda uurka ma tagtay mudada uurkan lahyd wali?? | - - - 1. Haa       2. Maya | **ugudu,S403** |  |
| S403 | Imisa gor ayad tagtay Daryeelka,Urkan dhaxdis | Tirada ______ |  |  |
| S404 | Mudadii uurkan dhaxdiisa ma lagu gu sheegay calamdo cudurka dhiig kara | - - - 1. Haa       2. Maya |  |  |
| S405 | Waligaa malagugu sheegay cudurka macaanka ama sonkrowga? | - - - 1. Haa       2. Maya |  |  |
| S406 | Wax dhaawac ama shil ah ma lakulantay mudadan uurka? Tusale;Shil baabuur IWM. | - - - 1. Haa       2. Maya   88. Ma xuusto |  |  |
| S407 | Waa imasa cabirka dhererka hooyada? | ______Dherarka oh SM |  |  |
| S408 | Wax khamri ah ma cabtay urkan dhaxdiisa? | - - - 1. Haa       2. Maya   88. Ma xuusto |  |  |
| S409 | Ilaa xilima ayaad khamrida cabaysay? Hal cadad ah waxay udhigantaa  1, Hal galas oo beer, wayn,Tella,Tej bordi  2, Hal galas oh ARAKE,JIIN,WISKI,Iwm. | - - - 1. Maalinkasta       2. 5-6 Jeer Todobaadkiiba       3. 3-4 jeer todobaadkiba       4. 1 jeer todobaadkiiba       5. Bishi hal mar       6. 3-8 jeer mudadii uurkan       7. 1-2jeer mudadi uurkan t  1. Ma xusuusto |  |  |
| S410 | Waligaa jaad maqayishay adoo uurleh? | - - - 1. Haa       2. Maya   88. Ma xusuusto |  |  |
| S411 | Ilaa xilimaad qayilaysay jaadka? | - - - 1. Maalinkasta       2. 5-6jeer todobaadkiiba       3. 3-4 jeer todobaadkiba       4. 1 jeer todobaadkiiba       5. Hal mar       6. 3-8 jeer mudadii Uurkan       7. 1-2 jeeer mudadii uurkan   88. Ma xuusto |  |  |

**Qaybta 5:- Xogta dhalmada kadib buuxi(Fiiri)**

*FG: Foomkan waa mid labuuxinayo kadib dhalmada adoo arkaya*

| **Tir** | **Su’aasha** | **Dhawrida** | **Ugudub** |
| --- | --- | --- | --- |
| **S501** | Hab nooce ah ayay ku umushay hooyadu? | - - - 1. Dhalmo caadi kutimid       2. Hooyo ku dhashay si caadiya laakin lagu gargaaray jeexdin yar oo xubinka taranka       3. Hooyo lagaga dhaliyay qalabka dumarka lagaga umuliyo       4. Hooyo lagaaga umuliyay qalitaan calaasha ah | **ugudu,S504** |
| **S502** | Mudo intee ah ayay qaadatay foosha heerka labaad? | Daqiidaha _______ |  |
| **Q503** | Hooyadu ma leedahay wax dilaaca dhanka xubinta?Does the women have perineal tear? | - - - 1. Haa       2. Maya |  |
| **S504** | Dhalmada kadibWax dhiig baxa ma leedhay hooyadu? | - - - 1. Haa       2. Maya | **ugudu,S506** |
| **S505** | Cadad ka dhiiga kabaxay hooyada | _______Qiyaastii ML |  |
| **S506** | Fooshu may adkayd? | 1. Haa 2. Maya |  |
| **S507** | Ilmo sidee ah | 1. Ilma nool 2. Todaba kudhalow |  |
| **S508** | Miisanka ilma oo giraam ah? **Fadlan ka tix raac faylka** |  |  |

Waan ku mahad celainayaa inaad ka qayb qadato

Qofka warystaha:

Magaca ____________________________Saxeexa _____________Tarikhda [___/___/___]

Masuulka hubiyay:

Magaca ____________________________ Saxeexa _________ Tarikhda [___/___/___]
